# Psychological consequences and the related factors among COVID‐19 survivors in southeastern Iran

**DOI:** 10.1002/hsr2.755

**Published:** 2022-08-10

**Authors:** Esmat Amiri Gooshki, Parvin Mangolian Shahrbabaki, Neda Asadi, Mahin Salmani

**Affiliations:** ^1^ Nursing Research Center, Department of Critical Care Nursing Kerman University of Medical Sciences Kerman Iran; ^2^ Department of Mathematics and Statistics University of New Brunswick Fredericton Canada

**Keywords:** COVID‐19, Iran, patient, psychological consequences

## Abstract

**Introduction:**

Coronavirus disease‐2019 (COVID‐19) is a new viral disease that has spread rapidly worldwide since December 2019 and there is no effective treatment for it. The current study aimed to investigate the psychological consequences and related factors among COVID‐19 survivors.

**Methods:**

This descriptive analytical study was conducted on 152 patients with COVID‐19 referred to referral hospitals in southeastern Iran in 2020. Data collection tools were three questionnaires of demographic and background information, Depression Anxiety stress Scale (DASS‐21) and Impact of Events Scale‐Revised (IESR). Descriptive and inferential statistics and SPSS25 were used to analyze the data.

**Results:**

The mean age of patients was 39.52 ± 13.16 years. The patients were mostly female (63.8%). Seventy‐three percent of the patients had severe posttraumatic stress disorder, 26.3% had moderate depression and 26.3% had severe anxiety. The mean scores of posttraumatic stress, depression, and anxiety among patients with COVID‐19 were 41.59 ± 17.28, 12.13 ± 9.16, and 12.45 ± 10.71, respectively. Intensive care unit (ICU) admission, divorce, illiteracy, and retirement were all associated with higher psychological load among patients.

**Discussion and Conclusion:**

The results showed that patients with COVID‐19 had different levels of anxiety, depression, and posttraumatic stress. These results may direct the attention of the medical staff to the mental health of COVID‐19 patients, necessitating timely psychological care and intervention during an epidemic.

## 1 INTRODUCTION

Coronavirus disease‐2019 (COVID‐19) is a new viral disease that has spread rapidly worldwide since December 2019, with severe acute respiratory syndrome coronavirus 2 (SARS‐CoV‐2) as its primary cause.^1^ The disease manifests itself in a variety of ways, ranging from very mild symptoms to acute respiratory distress syndrome and death.^2^ Approximately 144,358,956 cases with COVID‐19 and 3066113 deaths were recorded worldwide on April 23, 2021.^3^ COVID‐19 is a special and rare condition that is rapidly transmitted from person to person through respiratory droplets. Symptoms typically include cough, fever, shortness of breath, tiredness, muscle pain, lung infection, lymphocytopenia, and loss of taste or smell.^4^

What makes COVID‐19 so prevalent is the absence of effective treatment, the severity of the risk, the unpredictability of the condition, and the confusion surrounding when to control the disease. Quarantine and social restrictions are far more stringent than in similar circumstances due to concerns about the rapid transmission of the virus to others.^5^ People under quarantine lose personal and social communication, as well as traditional and even religious ceremonies, which can be stressful and lead to resentment and loneliness. These challenges in combination with some disinformation, exaggeration of risks, or suspicion about public health and personal safety increase anxiety and contribute to psychological disorders such as stress, anxiety, and depression. Paying attention to psychological issues in the face of the COVID‐19 crisis is an important part of health, as long‐term increases in cortisol levels and sympathetic stimulation will be detrimental, leading to a weakened immune system and a decreased body's ability to fight diseases, including COVID‐19.^6^, ^7^

In India, a study investigated the psychological impact of COVID‐19 disease. The results showed that a rapid increase in fear and anxiety due to closures and quarantines could lead to serious psychological disorders among these patients.^8^ According to a study conducted in China, 10.8% of the COVID‐19 patients showed posttraumatic stress disorder.^9^ Kong et al. in China discovered that 4.86% of the COVID‐19 patients experienced severe anxiety and 13.89% experienced mild depression.^10^

Unfortunately, this virus has spread to Iran and other countries in the world, and the fight against it is being conducted nationwide. According to the report from Iran's Ministry of Health, Treatment and Medical Education, the total number of patients with COVID‐19 was 2,438,193, the total number of deaths was 70,532 and 1,907,190 patients were recovered in the first week of May 2021.^11^ Due to the virus pathogenicity, the rate of spread as well as the mortality rate, this disease may compromise the mental health of individuals at different levels of society.

Given the wide range of stressors among survivors of infectious diseases, it seems that their stress does not decrease over time and may intensify, having a severe impact on their quality of life, function, and mental health.^12^ As COVID‐19 continues to spread, and given its psychological consequences as well as its emergence, the current study aimed to determine the psychological consequences and related factors among COVID‐19 patients referred to hospitals in southeastern Iran in 2020.

## 2 METHODS

### 2.1 Study design and setting

This descriptive‐analytical study investigated the psychological consequences and related factors among COVID‐19 survivors referred to referral hospitals in southeastern Iran.

### 2.2 Sample and sampling

The study population was all patients with COVID‐19 referred to referral hospitals who were selected through convenience sampling. According to the literature review, the correlation coefficient between depression and anxiety (0.512) in previous studies,^10^ the 99% confidence and 90% test power as well as taking into account the effect factor of 1.5 and the probability of dropout, 152 patients with COVID‐19 were included. Inclusion criteria were patients aged at least 18 years old, with normal speech, vision, and hearing, who had been sick for at least 1 month. Failure to complete more than one‐third of the questionnaire and psychological crises such as the death of a loved one or the birth of a child were considered as exclusion criteria.

### 2.3 Instruments

Data collection tools were three questionnaires: demographic and background information, Depression Anxiety Stress Scale (DASS‐21), and Impact of Event Scale‐Revised (IESR). Demographic and background information questionnaire included age, sex, occupation, marital status, education, family life, history of addiction, other chronic diseases, how to acquire knowledge about COVID‐19, length of hospital stay, ward, infection of close relatives with COVID‐19, postdischarge quarantine at home or convalescent home and a history of readmission.

The DASS21 was developed by Lovibond and Lovibond in 1995^13^ to assess symptoms of stress,^5^, ^7^, ^10^, ^11^, ^13^, ^14^anxiety,^3^, ^6^, ^8^, ^15^, ^16^, ^17^ and depression.^2^, ^4^, ^9^, ^12^, ^18^, ^19^, ^20^ A score of 0–14 was considered as normal stress, 15–18 was considered as mild, 19–25 was considered as moderate, and 26–33 was considered as severe. A score of 0–9 was considered as normal depression, 10–13 was considered as mild, 14–20 was considered as moderate, and 21–27 was considered as severe. A score of 0–7 was considered as normal anxiety, 8–9 was considered as mild, 10–14 was considered as moderate, and 15–19 was considered as severe. These subscales are scored by the addition of the total item scores. Scoring was based on a four‐point Likert scale (never = zero and always = 3). Since DASS21 is the short form of the original scale (42 items), the total score of each subscale had to be doubled and the severity of the symptoms was calculated. The validity of the DASS21 in the Tran study was 0.77.^15^ Beck Depression Inventory, Zung Self‐Rating Anxiety Scale, and Perceived Stress Scale were used simultaneously to evaluate the scale validity. The correlation between depression subscale of the DASS and Beck Depression Inventory was 0.70, the correlation between anxiety subscale of the DASS21 and Zung Self‐Rating Anxiety Scale was 0.67, and the correlation between stress subscale of the DASS21 and Perceived Stress Scale was 0.49. Factor analysis confirmed the three‐factor structure of this questionnaire.^18^ Dahm measured the reliability of the questionnaire. First, Beck Depression Inventory (BDI) and Beck Anxiety Inventory (BAI) were presented to a large sample of students. The correlation between BAI and anxiety subscale of DASS was high (*r* = 0.81) and BDI was highly correlated with DASS depression subscale (*r* = 0.74).^19^

The Impact of Event Scale‐Revised (IESR) was developed by Weiss (1997) to assess the dimensions of posttraumatic stress and to measure mental distress for specific traumatic events. This 22‐item scale has three subscales of avoidance (items 5, 7, 8, 11, 12, 13, 17, and 22), intrusion^2^, ^5^, ^8^, ^17^, ^18^and hyperarousal.^3^, ^9^, ^13^, ^14^, ^15^, ^16^, ^19^, ^20^ This scale is rated on zero (not at all) to four (extremely). To get the score for each subscale, the scores of each item would simply be added together. To obtain the overall score of the questionnaire, a scoring range of 0–88 was considered with a score of 33 being the cutoff point. A score between 0 and 33 showed mild symptoms and a score above 34–88 showed severe posttraumatic stress symptoms. The Persian version of IESR had good internal consistency with Cronbach's alpha between 0.87 and 0.67 as well as good test‐retest reliability (excluding hyperarousal subscale in the intervention group) (*p* < 0.001, *r* = 0.8–0.98). There were significant correlation coefficients between different dimensions of GHQ 28 and IESR subscales (excluding intrusion), and the three‐factor solution in factor analysis explained 41.6% of the variance.^16^

### 2.4 Ethical considerations

After receiving the code of ethics from Kerman University of Medical Sciences (IR.KMU.REC.1399.270, No. 99000194), the researcher identified the patients who met the inclusion criteria and contacted them. First, the necessary explanations about the research, its objectives, and confidentiality of information were given to the research units and their informed consent was obtained. The questionnaires were then sent to them via WhatsApp.

### 2.5 Data analysis

Descriptive statistics (frequency, percentage, mean and standard deviation) were used to describe demographic characteristics and mean scores. Inferential statistics (Pearson correlation coefficient, independent *t*‐test, and analysis of variance) were used to determine the relationship between variables. In addition, a multiple regression test was used to determine more accurate relationships and predictors.

## 3 RESULTS

The present study examined 152 patients with COVID‐19. The mean age of patients was 39.52 ± 13.16 years. Most of the patients were female (63.8%), married (64.47%), employed (57.9%), and had a bachelor's degree (34.2%). Most of the patients lived with their families (94.1%) and had no history of addiction (91.4%) and no other chronic diseases (72.4%). Most of the patients obtained their information about the COVID‐19 from the media (46.7%). In addition, the majority of patients reported infection of close relatives with COVID‐19 (51.97%). A high percentage of patients did not need to be admitted (49.3%) and chose home as a quarantine location (92.1%). There was no history of readmission after discharge (96.7%) (Table 1).

**Table 1:** The relationship between demographic characteristics and psychological consequences among COVID‐19 survivors

			Posttraumatic stress		Anxiety		Depression	
Variable		Frequency (%)	Mean ± SD	*p*	Mean ± SD	*p*	Mean ±SD	*p*
Sex^a^	Male	55 (36.2)	2.72 ± 44.86	0.85	10.89 ± 13.83	0.13	9.4 ± 12.35	0.8
	Female	97 (63.8)	2.01 ± 45.51		10.58 ± 11.67		9.06 ± 12	
Marital status^b^	Single	30 (19.7)	4.35 ± 40.94	0.03	9.09 ± 10.41	0.001	9.61 ± 9.79	0.001
	Married	98 (64.47)	1.86 ± 43.86		10.31 ± 10.59		7.59 ± 10.96	
	Divorced	8 (5.26)	7.12 ± 60.75		6.76 ± 14		12.53 ± 15.75	
	Widowed	16 (10.52)	3.86 ± 53.46		10.71 ± 23.60		10.41 ± 19.6	
Education level	Uneducated	51 (33.55)	2.37 ± 55.83	0.001	9.52 ± 22.76	0.001	9.2 ± 19.36	0.001
	Middle school	16 (10.5)	6.33 ± 42.81		10.42 ± 10.75		11.56 ± 10.87	
	Diploma	16 (10.5)	4.66 ± 36		6.37 ± 6.74		5.62 ± 8.44	
	Associate degree	6 (3.9)	1.26 ± 42		5.21 ± 4		3.2 ± 8.33	
	Bachelor's	52 (34.2)	2.30 ± 38.07		6.13 ± 6.69		5.17 ± 7.54	
	Master's	11 (7.2)	7.57 ± 49.27		5.67 ± 8.73		7.1 ± 10.55	
Job^b^	Self‐employed	14 (9.2)	6.15 ± 50.07	0.09	12.11 ± 12.57	0.28	12.41 ± 13.43	0.34
	Employed	88 (57.9)	2.02 ± 44.95		11.08 ± 10.5		7.87 ± 10.9	
	Retired	8 (5.3)	8.74 ± 56.37		8.77 ± 12.25		10.6 ± 17.75	
	Housewife	32 (21.1)	3.33 ± 43.46		12.09 ± 13.25		9.75 ± 12.87	
	Worker	10 (6.57)	10.41 ± 33.2		9.42 ± 15.8		10.93 ± 14.2	
Family life^b^	Yes	143 (94.1)	1.60 ± 44.33	0.02	10.74 ± 12.13	0.06	8.77 ± 11.92	0.24
	No	9 (5.9)	8.45 ± 60.22		9.48 ± 17.05		13.85 ± 15.15	
Addiction history^a^	Yes	13 (8.6)	7.99 ± 55.76	0.18	11.68 ± 17.69	0.06	13.15 ± 16.77	0.72
	No	139 (91.4)	1.58 ± 44.29		10.52 ± 11.96		8.63 ± 11.69	
Other chronic diseases^a^	No	110 (72.4)	1.84 ± 45.59	0.75	10.69 ± 12.58	0.81	8.9 ± 11.71	0.42
	Yes	42 (27.6)	3.29 ± 44.45		10.87 ± 12.10		9.84 ± 13.27	
Acquisition of information	Media	71 (46.7)	2.34 ± 44.80	0.95	11.13 ± 13.54	0.43	10.04 ± 13.18	0.21
	Internet	57 (37.5)	2.86 ± 45.40		10.08 ± 11.23		7.59 ± 10.49	
	Others	24 (15.8)	3.37 ± 46.37		11.02 ± 12.25		9.63 ± 12.92	
Close relatives' infection with COVID‐19	Yes	79 (51.97)	2.09 ± 45.37	0.95	10.31 ± 10.33	0.36	9.13 ± 11.89	0.76
	No	73 (48.02)	2.49 ± 45.17		10.28 ± 10.84		9.24 ± 12.38	
Ward type^b^	Not admitted	75 (49.34)	2.12 ± 42.92	0.01	9.35 ± 10.54	0.001	11.22 ± 41	0.01
	Infectious	62 (40.78)	2.47 ± 44.43		11.14 ± 12.29		8.66 ± 8.73	
	ICU	15 (9.86)	8.65 ± 64.44		10.10 ± 22.67		10.64 ± 11.66	
Quarantine place^a^	Home	140 (92.1)	1.68 ± 45.44	0.73	10.25 ± 10.70	0.13	9.19 ± 12.19	0.82
	Convalescent home	12 (7.9)	5.94 ± 43.33		10.76 ± 9.16		9.14 ± 11.5	
Post‐discharge readmission^a^	Yes	5 (3.3)	8.82 ± 61.4	0.07	8.83 ± 12	0.3	7.6 ± 13.25	0.51
	No	147 (96.7)	1.62 ± 44.73		10.33 ± 10.53		9.23 ± 12.08	

Abbreviations: ANOVA, analysis of variance; COVID‐19, coronavirus disease‐2019; ICU, intensive care unit; SD, standard deviation.

a Independent *t*‐test.

b ANOVA.

The mean scores of posttraumatic stress, depression, and anxiety among COVID‐19 survivors were 41.59 ± 17.28, 12.13 ± 9.16 and 12.45 ± 10.71, respectively. Seventy‐three percent of the COVID‐19 survivors had severe posttraumatic stress, 26.3% had moderate depression and 26.3% had severe anxiety (Table 2).

**Table 2:** Mean (SD) scores of posttraumatic stress, depression, and anxiety of COVID‐19 survivors

Variable	Min	Max	Mean	SD
Posttraumatic stress	0	84	41.59	17.28
Depression	0	40	12.13	9.16
Anxiety	0	38	12.45	10.71

Abbreviations: COVID‐19, coronavirus disease‐2019; SD, standard deviation.

There was a significant difference between the posttraumatic stress, marital status, level of education, family life, and type of ward. The mean score of posttraumatic stress was higher among the COVID‐19 patients who were divorced, illiterate, were not living with family members, and admitted to the ICU. There was a significant difference between the mean anxiety, marital status, level of education, and type of ward. Therefore, the mean anxiety was higher among patients who were widowed, illiterate, and admitted to ICU. There was a significant difference in the mean depression, marital status, level of education, and type of ward among patients with COVID‐19. The highest mean score of depression was related to patients who were married, widowed, and illiterate. In addition, patients admitted to the ICU showed a higher mean score of depression, which was significantly different from patients not admitted or admitted to the infectious ward (Table 1).

Other results of this study include factors related to the psychological consequences of COVID‐19 survivors. ICU admission, divorce, illiteracy, and retirement were some of the factors associated with higher psychological load among patients and showed more posttraumatic stress, anxiety, and depression. Table 3 showed that among the predictors affecting stress, variables of education level and family life had a greater effect on the stress of COVID‐19 survivors. A coefficient of determination of 0.142 indicated that 14% of the variances in stress were a function of mentioned predictors. Among the predictors affecting anxiety and depression, the variables of marital status, level of education, and age had the greatest effect on the anxiety and depression among the COVID‐19 survivors. The coefficients of determination of 0.565 and 0.551 indicated that 56% and 55% of the variances in anxiety and depression, respectively, were a function of mentioned predictors (Table 3).

**Table 3:** Analysis of regression of predictors of depression, anxiety and posttraumatic stress among COVID‐19 patients

Predictors		Raw			Adjusted		
		Regression coefficient	Coefficient of determination	*p* value	Regression coefficient	Coefficient of determination	*p* value
Stress	Marital status	4.25	0.049	0.006	2.29	0.142	0.161
	Education level	−3.17	0.089	>0.001	−2.45		0.008
	Family life	14.47	0.031	0.031	14.87		0.021
	Ward type	5.04	0.038	0.016	1.99		0.348
Anxiety	Marital status	3.12	0.165	>0.001	1.54	0.565	0.002
	Education	−3.30	0.508	>0.001	−2.88		>0.001
	age	0.109	0.031	0.031	−0.121		0.002
	Addiction	−4.55	0.026	0.048	−2.61		0.12
	Ward	3.15	0.092	>0.001	0.699		0.288
Depression	Marital status	3.09	0.167	>0.001	1.55	0.551	0.002
	Education	−2.91	0.488	>0.001	−2.71		>0.001
	Age	0.112	0.034	0.023	0.112		0.005
	Addiction	−4.96	0.032	0.027	−2.93		0.08
	Ward	3.30	0.105	>0.001	0.879		0.180

Abbreviation: COVID‐19, coronavirus disease‐2019.

## 4 DISCUSSION

The mean score of posttraumatic stress in the present study was 41.59 ± 17.28. According to the questionnaire cutoff point,^21^ the COVID‐19 survivors in this study experienced severe posttraumatic stress. According to the DASS‐21, the mean score of depression among these individuals was 12.13 ± 9.16, indicating mild depression. In addition, the mean score of anxiety was 12.10 ± 45.71, suggesting moderate anxiety. Mohammadi et al. showed that the mean scores of stress, anxiety, and depression among patients with COVID‐19 were 28.5 ± 59.18, 27.5 ± 62.12, and 28.5 ± 07.06, respectively.^17^ Vlake et al. found that the mean score of posttraumatic stress disorder among patients with COVID‐19 was 9 one month after discharge. In addition, the mean scores of anxiety and depression were four and five, respectively.^20^ Psychological symptoms among COVID‐19 survivors may be due to a variety of factors, including high COVID‐19 mortality rate and long‐term quarantine.^22^ Epidemics, on the other hand, never affect the entire population equally.^23^ Furthermore, most ordinary people get their information about COVID‐19 from social media, which frequently exaggerates the disease's implications and causes panic and anxiety among patients.^24^ Additionally, as the unknown disease progressed and there was a lack of suitable treatment in the early stages of COVID‐19, patients' fear of survival intensified. Anxiety and psychosis have been identified as two major mental health problems among patients with COVID‐19.^25^ However, the differences between the present study and related studies may be due to changes in the study populations' sociodemographic characteristics, follow‐up intervals, and tools for assessing the psychological consequences of patients with COVID‐19.

In the present study, 73% of the COVID‐19 survivors experienced severe posttraumatic stress. In addition, 26.3% had moderate depression and 26.3% had severe anxiety. Salimi et al. showed that 43.1% and 12.8% of the patients with COVID‐19 experienced postdischarge anxiety and depression, respectively. Furthermore, most of the patients experienced some stress (60.6%).^26^ Mak et al. reported that 15.6% of the patients with acute respiratory syndrome had depression.^27^ Wu et al. demonstrated that 13.5% of the patients with COVID‐19 developed anxiety and 10.8% of them developed depression.^24^ Vlake et al. found that 16% of the patients with COVID‐19 experienced posttraumatic stress disorder 1 month after discharge. Moreover, 29% of the patients reported potential anxiety and 32% reported potential depression 1 month of discharge.^20^ Kong et al. indicated that 34.72% and 28.47% of the patients with COVID‐19 had symptoms of anxiety and depression, respectively. They showed that 17.36% of the patients had mild anxiety, 12.5% had moderate anxiety and 4.86% had severe anxiety. They also reported that 13.89% had mild depression, 10.42% had moderate depression and 4.17% had severe depression.^10^ Fear and anxiety increased dramatically among COVID‐19 patients in India, according to Sood, due to the uncertainty of illness treatment. Additionally, simple but socially disruptive measures such as closure and quarantine can lead to serious psychological disorders such as posttraumatic stress disorder, depression, and anxiety.^8^ Given a wide range of stressors experienced by survivors of infectious diseases, it seems that their stress does not only persist but may also increase over time, resulting in detrimental effects on their quality of life, function, and consequently mental health. Therefore, control measures and psychological interventions are necessary to help COVID‐19 survivors improve their mental health.

According to the results of the present study, the mean difference of posttraumatic stress was significant in terms of marital status, level of education, family life, and type of ward. The mean score of posttraumatic stress was higher among patients with COVID‐19 who were divorced, illiterate, or did not live with a family. Liu et al. showed that the symptoms of posttraumatic stress disorder were significantly associated with high levels of loneliness among COVID‐19 patients.^28^ In the current study, patients admitted to the ICU had higher level of posttraumatic stress. Vlake et al. found no difference in the severity of posttraumatic stress symptoms between ICU and non‐ICU patients.^20^ Wang et al. reported high levels of stress in women and students.^29^ Salimi et al. showed a significant relationship between stress, the number of children and length of hospital stay in patients with COVID‐19.^26^ Post‐discharge respiratory symptoms, gender, and concerns about recurrence and transmission of the disease to others were among determinants of adverse psychological conditions among COVID‐19 survivors.^24^ However, the widespread, diverse, and negative psychological effects of COVID‐19, such as posttraumatic stress disorder, can lead to prolonged quarantine, fear of re‐infection with COVID‐19, frustration, lack of basic requirements, and insufficient information as well as economic problems. Patients admitted to the ICU may be more concerned about what will happen to them, so they are more stressed. In addition, previous studies have identified ICU patients as having a significant risk of psychological effects.^30^, ^31^, ^32^

There was a significant difference in the mean depression, marital status, level of education, and type of ward among COVID‐19 survivors. Patients who were widowed or uneducated had the highest mean depression. According to Vahedian‐Azimi et al., the rate of depression among single individuals was substantially greater than that of the married group.^21^ Kong et al. found that people with less social support were more depressed. Depression was associated with age, family members' infection with COVID‐19, and social support.^10^ Wu et al. showed a significant relationship between depression, female gender, post‐discharge respiratory symptoms, concern about recurrence and transmission of infection to others, and home quarantine.^24^ Home quarantine, on the other hand, might cause boredom and in some circumstances depression. Although the coronavirus brings families together, they may develop posttraumatic stress disorder, anxiety, and depression throughout the quarantine period.^33^

In the present study, there was a significant difference in mean anxiety, marital status, level of education, and type of ward among patients. The mean anxiety was higher in widowed, uneducated, and ICU patients. Vahedian‐Azimi et al. showed a relationship between anxiety and gender among COVID‐19 patients, so men had more anxiety than women.^21^ Mohammadi et al. showed higher anxiety scores among men in the whole population (people with COVID‐19 and healthy people) compared to women. In addition, the anxiety of COVID‐19 patients with mental illness was significantly higher than that of patients without mental illness.^17^ Wang et al. indicated that up‐to‐date and accurate health information (such as disease treatment and prevalence) and specific precautions (such as hand hygiene and wearing a mask) could reduce stress, depression, and anxiety.^29^ Liu et al. reported a significant association between clinical levels of anxiety and high levels of loneliness and concern about COVID‐19.^28^ The coronavirus, on the other hand, is a source of concern in the current scenario and requires special attention and care. It is normal for people to experience anxiety and fear, especially when they feel threatened and do not have the ability to deal with it.

## 5 CONCLUSION

The results of this study showed that COVID‐19 survivors had severe anxiety, moderate depression, and severe posttraumatic stress disorder. These results may draw the attention of the medical staff to the mental health of patients with COVID‐19, and timely psychological care and intervention may be required for patients during an epidemic to maintain individuals' mental health with appropriate psychological strategies and techniques.

## TRANSPARENCY STATEMENT

The lead author (Neda Asadi) affirms that this manuscript is an honest, accurate, and transparent account of the study being reported; that no important aspects of the study have been omitted; and that any discrepancies from the study as planned (and, if relevant, registered) have been explained.

## AUTHOR CONTRIBUTIONS

**Esmat Amiri Gooshki**: Conceptualization; formal analysis; writing—original draft. **Parvin Mangolian Shahrbabaki**: Conceptualization; methodology. **Neda Asadi**: Conceptualization; data curation; methodology; project administration; writing—original draft. **Mahin Salmani**: Conceptualization; formal analysis; methodology.

## CONFLICTS OF INTEREST

The authors declare no conflicts of interest.

## Data Availability

The authors confirm that the data supporting the findings of this study are available within the article [and/or] its supplementary materials.
